# Hospital Meetings, &c.

**Published:** 1897-12-11

**Authors:** 


### HOSPITAL MEETINGS, &C.

### SURGICAL AID SOCIETY.

The thirty-fifth annual meeting of the Surgical Aid Society was held at the Mansion House on Tuesday, the 7th inst., the Lord Mayor in the chair. Amongst those present were: Mr. Sheriff Dewar, Sir H. W. Peek, Bart., the Rev. Canon Barker, the Rev. Prebendary Kitto, the Rev. Walter Green, Mr. J. Herbert Tritton, and Mr. W. Gray (treasurer). The report which was read by the secretary stated that the nett expenditure during the year ended September 30th, 1897, had been £12,751, of which £10,304 was expended in relief, £1,939 on management expenses, and £507 in repayment of loan from bankers and interest. The income (including £1,240, legacies) amounted to £13,197. The number of patients assisted during the year was 14,550, *i.e.*, 5,217 men, 7,871 women, and 1,462 children, to whom 22,247 appliances had been given. Three new provincial branches have been formed (one at Plymouth, one at Cardiff, and one for Bristol and Bath), making the total number of branches 17. The Lord Mayor, in moving the adoption of the report and accounts, said that the society was to be congratulated upon the progress made during the past year, especially when one looked to the fact that so many institutions had suffered in income during the past year from the very many calls which had been made upon everyone. He suggested that it would be much better if something could be done by the society to render it unnecessary for more than one letter to be required for each instrument. The Rev. Prebendary Kitto seconded, and the report and accounts were adopted. The re-election of the committee and other officials, and votes of thanks to the treasurer, the committee, the surgeons, and the Lord Mayor, concluded the business. Contributions amounting to £452 were announced during the course of the meeting.

### THE HOSPITAL FOR SICK CHILDREN.

Owing to the proposal of the Masters of the Hospital of St. John and St. Elizabeth to build a new hospital on their property which would affect the light and air of the Hospital for Sick Children, Great Ormond Street, W.C., besides interfering with the utility of their out-patient operation-room and dispensary, a Special Court of Governors of the Great Ormond Street Hospital was held in their board-room on Wednesday, the 8th inst., to consider the advisability of purchasing the adjoining property, on which the Hospital of St. John and St. Elizabeth stands. Viscount Gort presided, in the absence of the Duke of Fife, who wrote regretting that, being obliged to leave London, he would be unable to take the chair. He added that he thoroughly appreciated the advantages of the scheme, which would add immensely to the usefulness of the hospital, and he therefore hoped it would be sanctioned by the governors.

Mr. Arthur Lucas, chairman of the Committee of Management, moved that an offer be made on behalf of the hospital to purchase the adjoining premises of the Hospital of St. John and St. Elizabeth for £30,000. The matter was first brought to the notice of the committee in 1893, when they were informed by the authorities of St. John and St. Elizabeth Hospital that it was intended to build a new hospital adjoining the Great Ormond Street Hospital. Very anxious and careful consideration was given to the matter, and negotiations which had ever since been going on had resulted in a sum of £30,000 being fixed as the price of the freehold. The purchase of this property, besides preventing an interference with the sanitary condition of the hospital, would enable the authorities to improve the accommodation for the nurses (the present arrangements being unhealthy and inconvenient) by housing forty of them in the convent building; would enable the authorities to rearrange some parts of the present building by taking advantage of this transference of the nurses; and would give the hospital a garden of about half an acre in addition to the garden at present rented from the Foundling Hospital. The committee had given grave and anxious consideration to the matter, and they thought that the proposed step was a wise and politic one to take.

Mr. J. Murray seconded, and Dr. Thomas Barlow (senior physician) and Mr. Edmund Owen (senior surgeon) supported the resolution, which was carried unanimously.

Mr. Justice Kekewich proposed, and Colonel Gordon Watson seconded, a resolution which provided that, in the event of the above offer being accepted, the £30,000 be provided by means of two loans of £10,000 and £20,000 on the best terms that could be arranged, to be secured as regards capital and interest by a charge on the invested general funds of the hospital and the purchased premises.

This resolution was agreed to, and it was decided that an appeal to the public for funds be made.

A vote of thanks to the chairman concluded the proceedings.

